# Biocompatibility of Mineral Trioxide Aggregate Mixed with Different Accelerators: an Animal Study

**DOI:** 10.30476/DENTJODS.2019.77826.0

**Published:** 2020-03

**Authors:** Mitra Tabari, Maryam Seyed Majidi, Mahtab Hamzeh, Saeideh Ghoreishi

**Affiliations:** 1 Dental Material Research Center, Dept. of Pediatric Dentistry, Babol University of Medical Sciences, Babol, Iran; 2 Dept. of Oral and Maxillofacial Pathology, Babol University of Medical Sciences, Babol, Iran; 3 Oral Health Research Center, Dept. of Pediatric Dentistry, Babol University of Medical Sciences, Babol, Iran; 4 Dental Material Research Center, Dept. of Pediatric Dentistry, Dental Research Institute, Isfahan University of Medical Sciences, Isfahan, Iran

**Keywords:** Biocompatibility, Mineral Trioxide Aggregate, Accelerator

## Abstract

**Statement of the Problem:**

Several additives have been introduced to decrease the setting time of MTA (mineral trioxide aggregate). For clinical applications, it is essential to investigate the biocompatibility of these materials.

**Purpose:**

The present study evaluated the tissue response to MTA that has been separately mixed with citric acid, calcium lactate gluconate (CLG), and Na_2_HPO_4_.

**Materials and Method:**

In this experimental study Twenty one Wistar rats were divided into three groups of 7, 14 and 30 days follow up periods. Sterile polyethylene tubes were subsequently filled with MTA separately mixed with distilled water, 0.1% citric acid, 0.43% calcium lactate gluconate (CLG) and 15% Na_2_HPO_4_ and afterwards implanted subcutaneously. Empty tubes were implanted as negative control. At the end of their respective periods, the animals were sacrificed by anesthetic overdose and a biopsy was performed. The inflammatory responses were scored, classified and statistically analyzed using Kruskal-Wallis and Man-Whitney tests. Statistical significance was defined as *p*< 0.05.

**Results:**

There was no significant difference between test groups in any time period after implantation but the mean values of inflammatory responses were significantly more than that of the negative control group (*p*> 0.05). The mean values of inflammatory responses were decreasing over time in all test groups. These values did not significantly differ in any group except the CLG and Na_2_HPO_4_ groups.

**Conclusion:**

The inflammatory responses induced by MTA mixed with citric acid and MTA mixed with Na_2_HPO_4_ were comparable to that of the control MTA. MTA mixed with CLG provoked a moderate-to-severe inflammatory response at 7 days after implantation, so further study is required before clinical application of this cement.

## Introduction

When the pulp is exposed by dental caries or trauma, vital pulp therapy becomes the procedure of choice to preserve the primary teeth until their natural shedding [ 1 - 2 ]. After removing the infected and inflamed coronal pulp, the vital, uninfected radicular pulp tissue is covered by formocresol [ 3 ], ferric sulfate [ 4 ] or MTA (mineral trioxide aggregate) [ 3 ].

There is no consensus about the best amongst the various published methods of pulpotomy [ 3 , 5 ]. Formocresol has been the most common pulp capping material used in the last six decades. In spite of systemic absorption of formaldehyde (the major component of formocresol), pulpotomy with formocresol has shown success rate of 97%. The mutagenic and carcinogenic effects of formocresol on the pulp have been the subject of many studies over the past 20 years [ 6 - 7 ]. 

In recent years, the use of MTA for pulp-capping has been proposed as an alternative to formocresol [ 8 ]. MTA is a white or gray powder consisting of fine hydrophilic particles of tricalcium silicate, tricalcium oxide, tricalcium aluminate, and silicate oxide. MTA has been used in vital pulp therapies, in repairing furcal and lateral perforations and as a root-end filling material during apical surgery, because of its biological properties [ 9 - 12 ]. MTA shows antimicrobial and dentinogenic effects on the pulp and preserves pulp integrity after pulp-capping or pulpotomy. A systematic study about pulpotomy with MTA in primary teeth has shown that this method results in a lower failure rate, less internal resorption, and leads to greater success [ 13 ]. 

Despite the favorable properties of MTA, it has some disadvantages. The major disadvantages are its long setting time (75 minutes to 72 hours), difficult handling, and high cost [ 14 - 15 ]. Many studies have focused on these limitations and several accelerators have been introduced to decrease the long setting time of MTA cement [ 15 - 20 ]. It has been reported that mixing MTA with additives such as Na_2_HPO_4_ [ 17 ], citric acid, and calcium lactate gluconate (CLG) [ 20 ] significantly decreases the setting time, although few studies have been conducted on the biocompatibility of these materials [ 15 , 19 , 21 - 22 ]. For clinical applications, it is essential to investigate the biocompatibility of new materials. The present study evaluated the biocompatibility of MTA mixed with the three different accelerators mentioned above.

## Materials and Method

This experimental study was approved by the Research Council of Babol University of Medical Sciences (ethics committee no.3326). The experimental process was performed in histopathology laboratory of Babol University of Medical Sciences, Babol, Iran. The subjects were 21 healthy male Wistar rats of equal age weighing 250 to 300g.  The rats had not been previously subjected to experimental studies and their subcutaneous dorsal tissues were normal. After inhalation anesthesia using chloroform in a desiccator chamber, each rat was further anaesthetized by intramuscular injection of 10% ketamine (Alfasan; Woerden, The Netherlands) and 2% xylazine (Alfasan; Woerden, The Netherlands). The dorsal skin was shaved and disinfected with 10% povidone iodine (Darupakhsh; Tehran, Iran). Five separate 15-mm incisions were made through the dorsal skin using a no. 15 scalpel. Pockets were prepared by undermining the incisions longitudinally for 20 mm. There were five experimental groups. 

In group 1, MTA (Angelous Industrial, Brazil) was mixed with distilled water as a positive control. In group 2, MTA was mixed with 0.1% citric acid. In group 3, MTA was mixed with 43.4% CLG. In group 4, MTA was mixed with 15% Na_2_HPO_4_ (Merk, Darmstad, Germany). Finally, in group 5, an empty tube as a negative control was employed

All solutions were mixed according to manufacturers’ instructions. Sterile polyethylene tubes 1.5 mm of inner diameters and 7 mm in length were filled with the test materials for groups 1-4. After shaving the dorsal part of the rats' body, 5 incisions (each one 15mm) were performed at least 3cm apart. One test tube of each group was implanted into each of the 5 subcutaneous pockets. After placement of the tubes, the incisions were sutured with 3-0 nylon sutures (Supa Medical Devices, Iran). 

The rats were then divided into 3 groups, each consisting of 7 rats, and maintained for periods of 7, 14 and 30 days. At the end of their respective periods, the animals were dispatched by administering a high dose of anesthetic and a biopsy was performed on a 2.5 mm diameter tissue surrounding each test tube. Adhering to the hygienic principles, the animals were buried in a special place provided before for such projects. After fixation of the resected tissue samples in 10% formalin, they were serially sectioned into 4-ϻm-thick samples and stained with hematoxylin and eosin. The sections were evaluated by a pathologist using blind analysis with a light microscope (Olympus BX41, Japan) using 10× and 40× objective lenses. The inflammatory responses after implantation were scored and classified according to previously established scoring system [ 23 ] as (0) for no reaction; absence of inflammatory cells, (1+) for mild reaction; presence of mild chronic inflammatory infiltrate or <25 eosinophilic or giant cells, (2+) for moderate reaction; moderate chronic inflammatory infiltrates or 25-150 eosinophilic or giant cells, and (3+) for severe reaction, intense chronic inflammatory infiltrate or >150 eosinophilic or giant cells [ 23 ].

Freidman and Wilcoxon tests were used to compare the histological differences between the test materials. The differences in inflammatory responses for the three time periods were examined using Kruskal-Wallis and Man-Whitney tests. Statistical significance was defined as *p*< 0.05.

## Results

The mean and standard deviation of inflammatory responses in different periods are shown in Table 1. There was no significant difference between experimental groups in any time period after implantation but the mean values of inflammatory responses of all experimental groups were significantly more than negative control group. This amount of difference in the mean inflammatory values is shown in Table 1. 

**Table1:** mean scores and standard deviation of inflammatory response after 7, 14 and 30 days and p Value for comparison of test groups and each group in different time periods.

	Time	7 days	14 days	30 days	*p* Value
Groups		mean±SD			
Negative Control		0.57±0.53^a^	0.43±0.53^a^	0.14±0.37^a^	0.26
MTA		1.71±0.48^b^	1.29±0.48^b^	1.00±0.57^b^	0.06
CA		1.86±0.37^b^	1.57±0.53^b^	1.43±0.53^b^	0.26
CLG		2.14±0.69^b^*	1.43±0.53^b^	0.86±0.69^b^*	0.01
Na_2_HPO_4_		1.86±0.69^b^*#	1.71±0.48^b^#	1.00±00^b^*	0.01
*p* Value		0.003	0.01	0.001	

Different letters show statistical differences in each column,

* shows statistical difference between 7 and 14 days,

# shows statistical differences between 7 and 30 days.

Different superscript letters (^ab^) indicate significant difference between groups (*p*< .05).

**Figure1:**
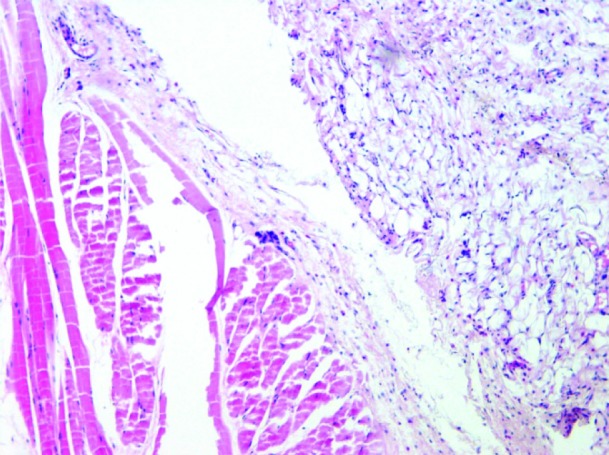
Grade 0 inflammation (negative control specimen), No inflammatory cells infiltration is seen in connective tissue and muscles

**Figure2:**
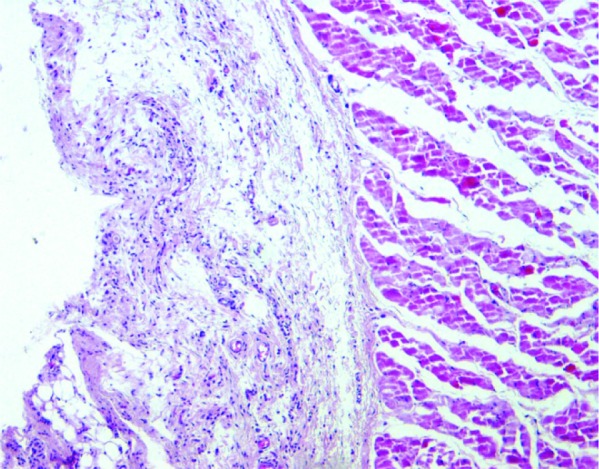
Grade 1+ inflammation (MTA specimen), Mild inflammatory cells are seen in connective tissue

**Figure3:**
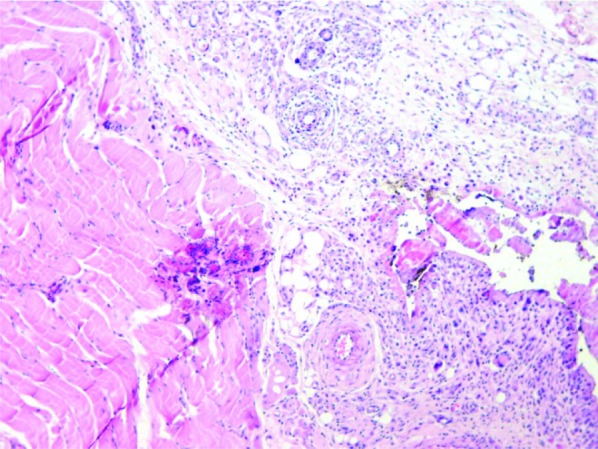
Grade 2+ inflammation (MTA specimen), Moderate infiltration of inflammatory cells is seen in connective tissue

**Figure4:**
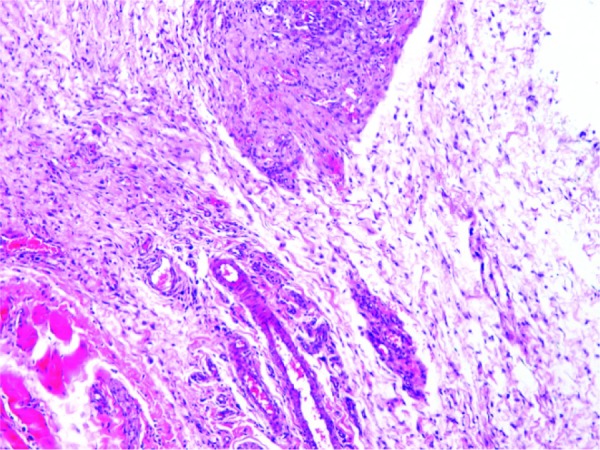
Grade 3+ inflammation (CLG specimen), Severe infiltrations of inflammatory cells are seen in connective tissue

**Figure5:**
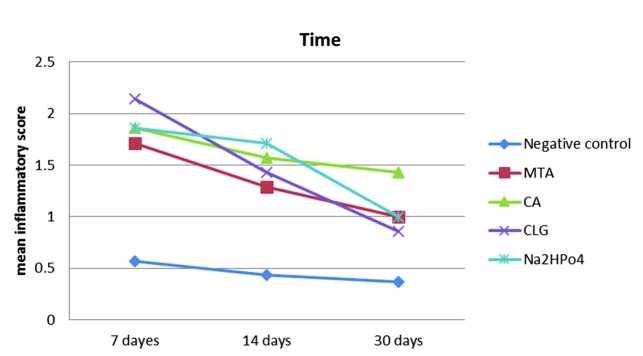
Mean score of inflammatory response of test groups in three time periods. (MTA= mineral trioxide aggregate, CA= citric acid, CLG= calcium lactate gluconate)

The mean values of inflammatory responses were decreasing over time in all test groups. These values were not statistically different in any group except groups 3 and 4. In group 3, the inflammatory response on the 7^th^ day after implantation was significantly more than that of the 30-days implantation (*p*= 0.01). In group 4, the inflammatory response seen at day 7 after implantation, was significantly more than that observed on 14^th^ and 30th days (*p*= 0.02). Photomicrographs of different inflammatory reactions of different experimental groups are presented in figures 1 to 4 and the mean inflammatory score of the test groups are shown in Figure 5. 

## Discussion

According to the results of this study, adding 0.1% citric acid, 43.4% CLG and 15% Na_2_HPO_4_ did not significantly affect the tissue response to MTA.

The biological evaluation of the potential risks of any new dental material is necessary before possible clinical application. Since a material in contact with vital tissues may have a destructive effect on the tissue, it is necessary to evaluate the degree and potential of any such effects of new materials to adjacent tissues. One way to evaluate the biocompatibility of such materials is to implant them in subcutaneous tissue and observe the inflammatory responses [ 24 ]. 

Subcutaneous implantation of materials in small laboratory animals can simulate in situ conditions of the material [ 25 ]. Mutoh *et al*. [ 26 ] reported that implanting materials using sterile tubes prevents the release of the substance into adjacent tissue. These tubes resemble a root canal of the tooth and are more advantageous than placing the material directly into the tissue. The inert nature of polyethylene tubes makes them suitable for implantation studies. In the present study, sterile polyethylene tubes were used for implantation. In this study, just as shown in previous studies [ 27 - 30 ], no reaction or mild inflammatory response decreasing over time was seen after implanting the empty negative control tubes. The initial inflammatory response to the empty tubes is considered a response to the surgical procedure of implantation [ 30 ]. 

On the 7^th^ day of follow-up period, MTA showed a mild to moderate inflammatory response that decreased over time; on the 30^th^ day of follow-up period, only a mild inflammatory response was observed. Similar to the results of this study, several studies on biocompatibility of MTA have reported that this cement initially causes a moderate inflammatory response that decreases over time [ 29 - 32 ]. The initial inflammatory response followed by MTA implantation can be explained by a response to pH, the heat generated during setting, and the production of inflammatory cytokines such as IL1 and IL6 in the beginning of the process [ 33 ].

In the present study, there were no significant differences between the inflammatory responses of MTA mixed with citric acid and the control MTA at any follow up periods. Low-dose citric acid (0.1%) has a neutral pH (pH = 7.35) and no cytotoxicity [ 34 ]. So citric acid was used in the present study at a very low concentration.

Despite the higher rate of inflammatory reaction to MTA mixed with citric acid, this mixture induced an acceptably mild-to-moderate response that decreased over time. Similar to the results of this study, Kang *et al*. [ 21 ] evaluated the cytotoxicity and the cellular response of MTA mixed with 0.1 % citric acid and reported a "good" response. In their study, this material showed a higher rate of cell viability than control MTA and the MTA mixed with Na_2_HPO_4_ at day 7 of experimentation. Lee *et al*. [ 35 ] reported that low-dose citric acid had no adverse effects on biocompatibility, osteogenic differentiation, and mineralization of MTA.

 CLG is a soluble salt of calcium, lactic acid and gluconic acid that is often used in effervescent calcium tablets. It is reported that this material can decrease the time-setting period of MTA to 13.9 minutes [ 20 ]. In the present study, no significant differences were seen between the histological response of this cement and the control MTA; however, analysis with more details showed an inflammatory reaction on the 7^th^ day that significantly differed from that of 30^th^ days. This cement showed moderate-to-severe inflammatory response (more than any other experimental groups) on day 7, which decreased to a mild reaction after 30 days (less than the others did). 

Ji *et al*. [ 36 ] performed a cytological study on the biocompatibility of MTA mixed with 23.1% CLG and reported that this cement led to greater cell viability than pure MTA. They said that this was attributed to minor differences but lower alkalinity of MTA/CLG. The difference between the results could be attributed to the difference in concentration of calcium gluconate used in the two studies. Kang *et al*. [ 21 ] concluded that, despite the higher survival rate of cells with MTA mixed with 43.4% CLG at 1 day, cell survival at 4 days and 7 days were significantly lower than that of pure MTA. They also observed that MTA mixed with 43.4% CLG released more calcium ions than did the mixture of MTA with distilled water. It appears that calcium ions released from MTA may positively affect the repair process as they can pass through the cell membranes by depolarization or activation of membrane-bound calcium channels [ 37 ]. On the other hand, high concentrations of intracellular calcium ions can cause cytotoxicity and trigger cell death [ 37 - 38 ]. Kang *et al*. [ 21 ] reported that lower cell viability for MTA mixed with CLG is likely a result of the high concentrations of free calcium ions. The CLG molecular weight used in this study was similar to that of Kang *et al*. [ 21 ]. This may explain the severe inflammatory response of this cement observed 7 days after implantation. Results of this study show that, despite the lack of a significant difference between the inflammatory responses of the control MTA and MTA mixed with CLG, the amount of inflammation in the early days cannot be overlooked. Similar to the results of the present study, Parirokh *et al*. [ 39 ] compared a combination of MTA with/without CaCl2 as pulp-capping agents in dogs' teeth and found a higher percentage of inflammation and necrosis and a lower percentage of calcified bridge formation in MTA/CaCl2 samples compared with MTA. Although the difference was not statistically significant; but, they concluded that addition of CaCl2 to MTA pulp-capping agent does not improve the properties of this biomaterial. Further studies on the biocompatibility of this cement are required before its clinical application. 

The inflammatory response of MTA mixed with Na_2_HPO_4_ showed no significant difference with that of the control MTA; however, a significant difference was observed between the histological responses induced by MTA mixed with Na_2_HPO_4_ 7 days and 14 days after implantation. This difference was also significant between 7^th^ and 30^th^ days, which indicates that this cement initially induced a mild-to-moderate inflammatory reaction (slightly more than MTA), but the intensity decreased significantly over time. On 30th day, aside from the negative control, the lowest inflammatory response between experimental groups was seen in this group.

Ding *et al*. [ 17 ] reported that mixing MTA with Na_2_HPO_4_ does not change the cell viability of pure MTA and this mixture is still biocompatible. They also found no changes in cell viability for white MTA mixed with Na_2_HPO_4_ at day 1 and day 7. Lotfi *et al*. [ 22 ] studied the effect of MTA mixed with 2.5% (wt) Na_2_HPO_4_ on inflammatory cells and reported that this material induced a mild inflammatory response at days 7, 15, 30 and 90. Similar to the results of present study, Kulan *et al*. [ 40 ] concluded that cell viability of MTA mixed with Na_2_HPO_4_ increases significantly over time. They concluded that adding Na_2_HPO_4_ produced more biocompatible cement. The higher inflammatory reaction seen in the present study might be explained by the higher molecular weight of the Na_2_HPO_4_ used; however, this cement induced an inflammatory response comparable to that of the control MTA.

At last, it should be kept in mind that mixing MTA with different accelerators may have adverse effects on physical properties especially compressive strength of the mixture. Different studies have reported varying results in this regard [ 41 - 43 ]. Further studies should be conducted to clarify the effect of accelerators on mechanical properties of MTA.

## Conclusion

For all of the accelerators tested in this study, the inflammatory response decreased over time. The results of this study indicate that the inflammatory responses induced by MTA mixed with 0.1% citric acid and MTA mixed with 15% Na_2_HPO_4_ were comparable to that of the MTA mixed with distilled water. These cements appear to be biocompatible within the limitations of this study. Despite the absence of significant differences between control MTA and MTA mixed with CLG for histological response, the latter cement provoked a moderate-to-severe inflammatory response on 7^th^ day after implantation. Further studies are suggested before clinical application of this cement.
